# Impact of an MT-RNR1 Gene Polymorphism on Hepatocellular Carcinoma Progression and Clinical Characteristics

**DOI:** 10.3390/ijms22031119

**Published:** 2021-01-23

**Authors:** Yang-Hsiang Lin, Yu-De Chu, Siew-Na Lim, Chun-Wei Chen, Chau-Ting Yeh, Wey-Ran Lin

**Affiliations:** 1 Liver Research Center, Linkou Chang Gung Memorial Hospital, Taoyuan 333, Taiwan; yhlin0621@cgmh.org.tw (Y.-H.L.); yudechu19871003@gmail.com (Y.-D.C.); chautingy@gmail.com (C.-T.Y.); 2 Department of Neurology, Linkou Chang Gung Memorial Hospital, Taoyuan 333, Taiwan; siewna.lim@gmail.com; 3 College of Medicine, Chang Gung University, Taoyuan 333, Taiwan; 4 Department of Hepatology and Gastroenterology, Linkou Chang Gung Memorial Hospital, Taoyuan 333, Taiwan; 8902088@cgmh.org.tw; 5 Molecular Medicine Research Center, Chang Gung University, Taoyuan 333, Taiwan

**Keywords:** hepatocellular carcinoma, mitochondrial DNA, MT-RNR1, hexokinase 2, prognostic predictor

## Abstract

Mitochondrial DNA (mtDNA) mutations are highly associated with cancer progression. The poor prognosis of hepatocellular carcinoma (HCC) is largely due to high rates of tumor metastasis. This emphasizes the urgency of identifying these patients in advance and developing new therapeutic targets for successful intervention. However, the issue of whether mtDNA influences tumor metastasis in hepatoma remains unclear. In the current study, multiple mutations in mtDNA were identified by sequencing HCC samples. Among these mutations, mitochondrially encoded 12S rRNA (MT-RNR1) G709A was identified as a novel potential candidate. The MT-RNR1 G709A polymorphism was an independent risk factor for overall survival and distant metastasis-free survival. Subgroup analysis showed that in patients with cirrhosis, HBV-related HCC, α-fetoprotein ≥ 400 ng/mL, aspartate transaminase ≥ 31 IU/L, tumor number > 1, tumor size ≥ 5 cm, and histology grade 3-4, MT-RNR1 G709A was associated with both shorter overall survival and distant metastasis-free survival. Mechanistically, MT-RNR1 G709A was clearly associated with hexokinase 2 (HK2) expression and unfavorable prognosis in HCC patients. Our data collectively highlight that novel associations among MT-RNR1 G709A and HK2 are an important risk factor in HCC patients.

## 1. Introduction

Increasing evidence indicates that mitochondrial DNA (mtDNA) mutations in both noncoding and protein-coding regions are highly correlated with disease and carcinogenesis, including in diabetes [1], obesity [2], Alzheimer′s disease [3,4], and cancer [5,6,7,8]. The mitochondrial genome is a 16,600 base-pair, closed-circular, double-helix that encodes 13 oxidative phosphorylation (OXPHOS) subunit proteins, 22 tRNAs, 2 rRNAs, and a displacement loop (D-loop) [9]. These proteins include seven subunits of complex I, one subunit of complex III, three subunits of complex IV, and two subunits of complex V. Mitochondrially encoded 12S rRNA (MT-RNR1) and 16S rRNA (MT-RNR2) are responsible for making rRNAs and are assigned to rRNA complexes [9]. Previous studies have indicated that *MT-RNR1 A1555G* mutation is positive correlated with hearing loss phenotypes [10,11]. Abril et al. demonstrated that the RNA levels of MT-RNR1, MT-CO2, and MT-ATP6 were downregulated in prostate cancer compared to paired normal tissue, suggesting that MT-RNR1 may be involved in prostate cancer progression [12]. Another study indicated that the expression level of MT-RNR1 may serve as a predictor for chemotherapy in ovarian cancer by multivariate analysis [13]. Those observations suggested that MT-RNR1 is associated with disease and cancer progression. A previous study reported that mitochondrially encoded ATP synthase membrane subunit (ATP6) was more susceptible to mutation in breast cancer, and this mutation may induce metabolic imbalances among cancer cells [14]. Another group demonstrated that a single-nucleotide variant in the mitochondrial NADH dehydrogenase (ND) gene was associated with distant metastasis in non-small cell lung cancer (NSCLC) and colon cancer [15]. Ishikawa et al. showed that mtDNA mutations regulate cancer cell metastasis via reactive oxygen species (ROS) signaling and the expression of ROS-sensitive genes [16]. Using cytoplasmic hybrid (cybrid) technology to replace endogenous mtDNA in a mouse cell line, the authors found that mtDNA contained G13997A and 13885insC mutations in the ND6 gene, which enhanced cell metastatic ability. Kulawiec and coworkers demonstrated that a mutation in tRNA Leu was associated with mitochondrial membrane potential, drug resistance, and metastasis using in vitro and in vivo assays [17,18]. Mechanistically, they found that mtDNA mutation enhanced the PI3K/AKT signaling pathway, thereby contributing to the induction of cancer cell metastasis. These findings suggest that alterations in both protein-coding and noncoding genes in mtDNA appear to be the most influential in inducing tumorigenesis.

Hepatocellular carcinoma (HCC) is an aggressive primary liver malignancy. In fact, the liver can adapt to facilitate energy homeostasis for the whole body, suggesting that metabolic reprogramming is an important event in HCC formation and progression. Cancer cells induce epithelial-mesenchymal transition (EMT)-related gene expressions, thus promoting tumor cell metastasis [19]. In general, the hallmark of tumor metastasis may contribute to a poor prognosis of cancer, including in HCC [20]. This indicates that novel biomarkers for monitoring cancer progression need to be identified. It has been reported that the low mtDNA copy number in HCC is significantly correlated with liver cirrhosis, large tumor size, and poor survival outcomes [21]. Several somatic mutations in mtDNA have been identified in HCC as well [22]. Although these mutations resulted in amino acid substitutions, no significant association was found between these mutations and the clinicopathological features of the HCCs. These observations suggest that noncoding regions/genes in mtDNA might be more important than protein-coding regions/genes.

In the current study, HCC specimens were collected and the whole mtDNA genome was sequenced by the Sanger method. Among these variations, some important mutations were identified in both noncoding regions and protein-coding regions. Furthermore, only the MT-RNR1 G709A was significantly correlated with overall survival and distant metastasis-free survival. These findings indicate that the MT-RNR1 (G709A) will be useful for effective diagnosis and prognosis or novel therapeutic target development for HCC.

## 2. Results

### 2.1. The MT-RNR1 (G709A) Polymorphism Is an Independent Prognostic Factor Associated with Survival in HCC

**Figure 1:**
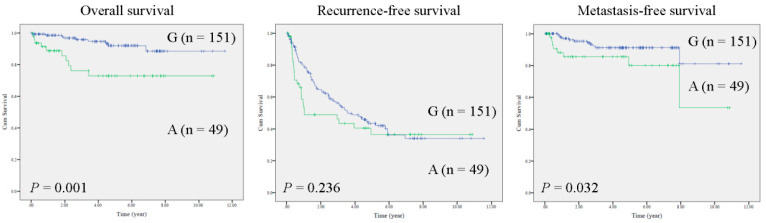
MT-RNR1 709A predicts poor prognosis in HCC. Kaplan–Meier analysis of survival outcome based on MT-RNR1 709G (G) or 709A (A) in HCC specimens (*n* = 200). Survival outcome was analyzed using the log-rank test.

In HCC tissues, the entire mtDNA was sequenced by the Sanger method in a training cohort (*n* = 25). Sequencing results were assembled and analyzed by bioinformatics tools. Notably, multiple mutations were identified in HCC specimens. Specifically, two mutations in noncoding regions (MT-RNR1 G709A and mtDNA D-loop 310insC) and three mutations in protein coding regions (MT-ATP6 A8710G, MT-ND3 A10398G and MT-ND3 C10400T) were identified (Supplementary Table S1). Furthermore, the survival functions of these mutations were calculated (*n* = 25) using the Kaplan–Meier method. Notably, only MT-RNR1 709A was correlated with poor prognosis in patients with metastatic HCC (extrahepatic metastasis) (Supplementary Figure S1). Accordingly, MT-RNR1 G709A was selected for further study. In total, the MT-RNR1 G709A polymorphism was present 24.5% of HCC specimens (49/200). To validate whether the MT-RNR1 G709A mutation was significantly correlated with survival outcome, a Kaplan–Meier analysis was performed in a validation cohort. As expected, MT-RNR1 709A was significantly associated with a shorter overall survival and distant metastasis-free survival in HCC patients but not with intrahepatic recurrence-free survival (Figure 1). However, MT-RNR1 709A was not correlated with any clinical parameters by Fisher’s exact test (Supplementary Table S2). In addition, univariate and multivariate analysis showed that MT-RNR1 G709A was an independent risk factor for overall survival (Supplementary Table S3) and metastasis-free survival (Supplementary Table S4) in HCC patients.

### 2.2. MT-RNR1 709A Is an Effective Prognostic Predictor in Subgroups of HCC

**Figure 2:**
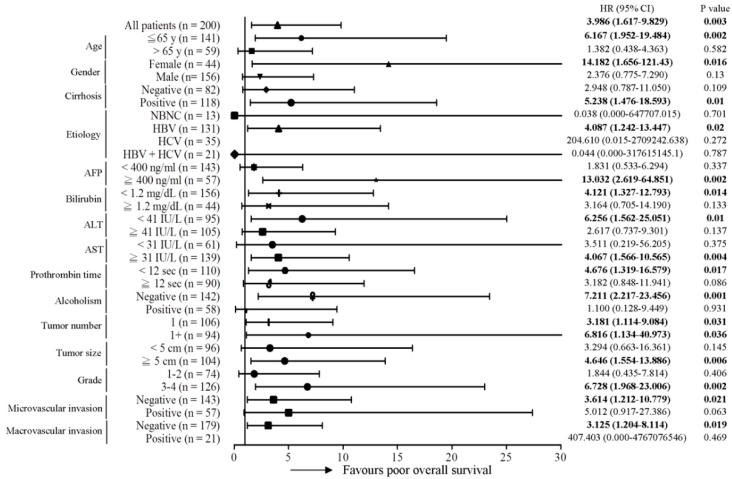
Forest plot subgroup analysis of HCC patients stratified by various clinical parameters to evaluate associations between MT-RNR1 G709A and overall survival. The Cox proportional hazard model was used to analyze MT-RNR1 G709A in relation to the overall survival in subgroups. Horizontal lines represent 95% confidence intervals. non-hepatitis B/non-hepatitis C (NBNC): patients without HBV or hepatitis C virus (HCV) infection. HBV: patients with HBV infection. HCV: patients with HCV infection. The cut-off values for AFP, ALT, and AST were 400 ng/mL, 41 IU/L, and 31 IU/L.

**Figure 3:**
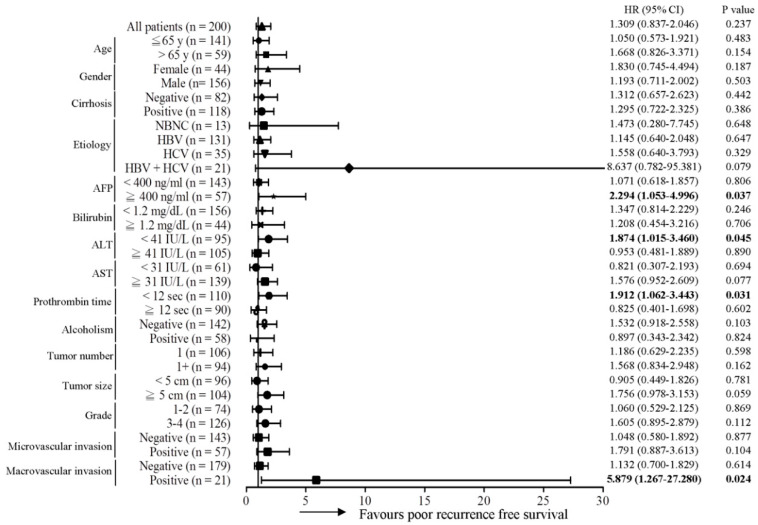
Forest plot subgroup analysis of HCC patients stratified by various clinical parameters to evaluate the association between MT-RNR1 G709A and intrahepatic recurrence-free survival. The Cox proportional hazard model was used to analyze MT-RNR1 G709A in relation to intrahepatic recurrence-free survival in subgroups. Horizontal lines represent 95% confidence intervals. NBNC: patients without HBV or HCV infection. HBV: patients with HBV infection. HCV: patients with HCV infection. The cut-off values for AFP, ALT, and AST were 400 ng/mL, 41 IU/L, and 31 IU/L.

**Figure 4:**
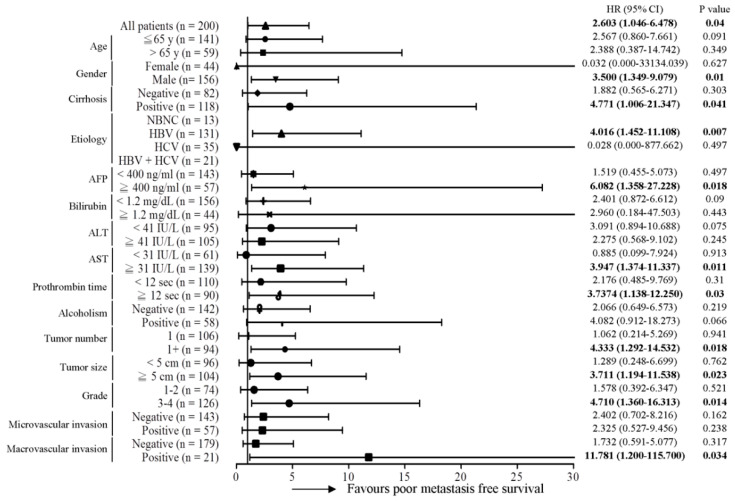
Forest plot subgroup analysis of HCC patients stratified by various clinical parameters to evaluate the association between MT-RNR1 G709A and distant metastasis-free survival. The Cox proportional hazard model was used to analyze MT-RNR1 G709A in relation to distant metastasis-free survival in subgroups. Horizontal lines represent 95% confidence intervals. NBNC: patients without HBV or HCV infection. HBV: patients with HBV infection. HCV: patients with HCV infection. The cut-off values for AFP, ALT, and AST were 400 ng/mL, 41 IU/L, and 31 IU/L.

Subsequently, the predictive role of the MT-RNR1 G709A mutation in various clinical subgroups was analyzed using the Cox proportional hazard method. Here, we performed a Forest plot analysis, revealing that MT-RNR1 709A was significantly associated with overall survival in the following subgroups: age ≤ 65 y (hazard ratio (HR) = 6.167, 95% CI 1.952–19.484, *p* = 0.002), female sex (HR = 14.182, 95% CI 1.656–121.43, *p* = 0.016), cirrhosis positivity (HR = 5.238, 95% CI 1.476–18.593, *p* = 0.01) hepatitis B virus (HBV)-related HCC (HR = 4.087, 95% CI 1.242–13.447, *p* = 0.02), alpha-fetoprotein (AFP) ≥ 400 ng/mL (HR = 13.032, 95% CI 2.619–64.851, *p* = 0.002), bilirubin < 1.2 mg/dL (HR = 4.121, 95% CI 1.327–12.793, *p* = 0.014), alanine transaminase (ALT) < 41 IU/L (HR = 6.256, 95% CI 1.562–25.051, *p* = 0.01), aspartate transaminase (AST) ≥ 31 IU/L (HR = 4.067, 95% CI 1.566–10.565, *p* = 0.017), prothrombin time (PT) < 12 s (HR = 4.676, 95% CI 1.319–16.579, *p* = 0.017), the alcoholism-negative group (HR = 7.211, 95% CI 2.217–23.456, *p* = 0.001), primary tumor (only 1 tumor) (HR = 3.181, 95% CI 1.114–9.084, *p* = 0.031), tumor number > 1 (HR = 6.816, 95% CI 1.134–40.973, *p* = 0.036), tumor size ≥ 5 cm (HR = 4.646, 95% CI 1.554–13.886, *p* = 0.006), histology grade 3-4 (HR = 6.728, 95% CI 1.968–23.006, *p* = 0.002), microvascular invasion negative (HR = 3.614, 95% CI 1.212–10.779, *p* = 0.021), and macrovascular invasion negative (HR = 3.125, 95% CI 1.204–8.114, *p* = 0.019) (Figure 2). Accordingly, MT-RNR1 709A was significantly correlated with overall survival in the subgroups of age ≤65 y, female sex, cirrhosis positivity, HBV-related HCC, AFP ≥ 400 ng/mL, bilirubin < 1.2 mg/dL, ALT < 41 IU/L, AST ≥ 31 IU/L, PT < 12 s, alcoholism-negative, primary tumor (only 1 tumor), tumor size ≥ 5 cm, histology grade 3-4, microvascular invasion negative, and macrovascular invasion negative group. Only four subgroups (AFP ≥ 400 ng/mL, ALT < 41 IU/L, PT < 12 s, and macrovascular invasion positive) of HCC were significantly associated with shorter intrahepatic recurrence-free survival (Figure 3). MT-RNR1 709A was also significantly correlated with distant metastasis-free survival in the following subgroups: male sex (HR = 3.500, 95% CI 1.349–9.079, *p* = 0.018), cirrhosis positivity (HR = 4.771, 95% CI 1.006–21.347, *p* = 0.041), HBV-related HCC (HR = 4.016, 95% CI 1.452–11.108, *p* = 0.007), AFP ≥ 400 ng/mL (HR = 6.082, 95% CI 1.358–27.228, *p* = 0.018), AST ≥ 31 IU/L (HR = 3.947, 95% CI 1.374–11.337, *p* = 0.011), PT ≥ 12 s (HR = 3.737, 95% CI 1.138–12.250, *p* = 0.03), tumor number > 1 (HR = 4.333, 95% CI 1.292–14.532, *p* = 0.018), tumor size ≥ 5 cm (HR = 3.711, 95% CI 1.194–11.538, *p* = 0.023), histology grade 3-4 (HR = 4.710, 95% CI 1.360–16.313, *p* = 0.014), and macrovascular invasion positivity (HR = 11.781, 95% CI 1.200–115.700, *p* = 0.034) (Figure 4). These findings indicated that MT-RNR1 709A was significantly associated with distant metastasis-free survival in the subgroups of male sex, cirrhosis positivity, HBV-related HCC, AFP ≥ 400 ng/mL, AST ≥ 31 IU/L, PT ≥ 12 s, tumor number > 1, tumor size ≥ 5 cm, histology grade 3-4, and macrovascular invasion positivity. Taken together, these findings indicated that shorter overall survival and metastasis-free survival preferentially occurred in patients with MT-RNR1 709A in some subgroups, especially in HBV-related HCC, AST ≥ 31 IU/L, and histology grade 3-4 patients (see Figure 2 and Figure 4, bold *p*-values).

### 2.3. The Impact of MT-RNR1 G709A on Hexokinase 2 and Mitochondrial Open-Reading-Frame of the Twelve S rRNA-c Expression

**Figure 5:**
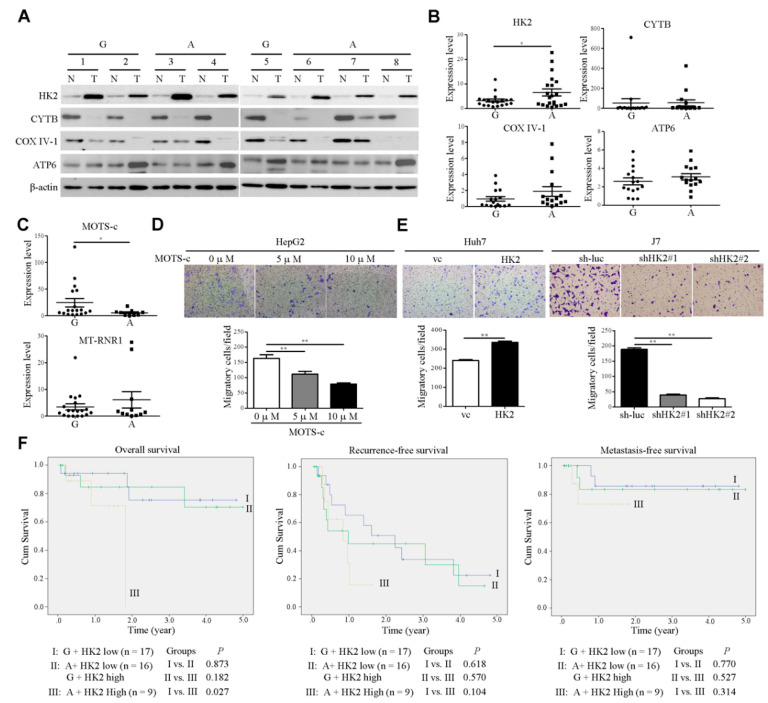
The MT-RNR1 709A/HK2 axis is correlated with poor prognosis in HCC patients. (**A**,**B**) Western blot analysis of HK2, CYTB, COXIV-1, and MT-ATP6 expression in paired HCC specimens. Protein expression was examined in eight representative pairs of HCC specimens, with β-actin as the loading control. The quantitative results of those genes were calculated and are shown in the MT-RNR1 709G and 709A groups. G: MT-RNR1 709G; A: MT-RNR1 709A. (**C**) Expression levels of MOTS-c and MT-RNR1 in HCC specimens were determined by qRT-PCR. 18S rRNA was used as a loading control. The transwell migration assay was performed in MOTS-c-treated cells (**D**) and HK2 stable cell lines (**E**). (**F**) Kaplan–Meier survival analysis curve of high- and low-risk survival groups in 43 paired HCC patients. MT-RNR1 709A and simultaneous high HK2 levels were significantly associated with the poorest overall survival. *p*-values were determined via the log-rank test. G: MT-RNR1 709G; A: MT-RNR1 709A. *, *p* < 0.05; **, *p* < 0.01.

The flexibility of changing the balance of glycolysis and OXPHOS, two forms of energy production, is a key event in cancer progression [23]. Cancer cells exhibit unique metabolic phenotypes through glycolysis and mitochondrial OXPHOS [24]. Both of these pathways provide considerable survival and metastatic advantages to cancer cells. Multiple studies have shown that glycolytic enzymes such as hexokinase 2 (HK2), which is involved in the first rate-limiting step of glycolysis, are associated with poor prognosis in several cancer types [25]. On the other hand, mitochondria function contributes to malignant transformation through the regulation of ROS production and oncometabolites [26]. To investigate whether MT-RNR1 G709A was associated with a glycolytic gene (HK2) or mitochondrial-related genes (CYTB, COXIV-1, and ATP6) in HCC, the protein levels of those genes were assessed using Western blot analysis. Notably, HK2 and ATP6 were highly upregulated in HCC compared to adjacent normal tissue (Figure 5A). In contrast, CYTB and COX IV-1 were downregulated in HCC compared to adjacent normal tissue (Figure 5A). Furthermore, only the HK2 expression levels but not the levels of other genes were significantly higher in the MT-RNR1 709A group than in the MT-RNR1 709G group (Figure 5B). These findings indicated that HK2 expression may be regulated by the MT-RNR1 709A polymorphism. Lee et al. found that MT-RNR1 can encode a small functional peptide, called the mitochondrial open reading frame, of the twelve S rRNA-c (MOTS-c) [27,28]. Notably, MOTS-c was identified to regulate disease and cellular metabolic functions [27,29]. Additionally, gene expressions were regulated by MOTS-c through the modulation of downstream signaling transduction, such as the MAPK/NFkB pathway [30]. Next, to figure out whether this functional peptide is also correlated with this polymorphism in hepatoma, the expression levels of MOTS-c were assessed using qRT-PCR. Meanwhile, the MT-RNR1 expression levels in HCC were also determined. The MOTS-c expression levels, but not those of MT-RNR1, were significantly lower in the MT-RNR1 709A group than in the MT-RNR1 709G group (Figure 5C). These observations suggest that the MT-RNR1 709A mutation regulates HK2 and MOTS-c expression.

Furthermore, the effect of HK2 and MOTS-c on motility in hepatoma cell lines was measured using a transwell migration assay. We found that MOTS-c treatment markedly reduced the cell migration of hepatoma cell lines (Figure 5D). The expression levels of HK2 in HK2-overexpressing Huh7 cell lines were measured using Western blot analysis (Supplementary Figure S2). On the other hand, infected HK2 #37672 (designated shHK2#1) and #196260 shRNA (designated shHK2#2) in J7 cell lines obviously repressed HK2 expression and were selected for further functional assay (Supplementary Figure S2). The overexpression of HK2 in Huh7 cell lines promoted cell migration (Figure 5E). Conversely, these phenotypes were reversed upon the knockdown of HK2 in J7 cell lines (Figure 5E). These findings suggested that MT-RNR1 709A disrupted the balance of glycolysis and OXPHOS, which then resulted in promoting cell migration. Because of the positive correlation between MT-RNR1 G709A and HK2 in HCC tumors, we evaluated their combined effects on survival outcomes in patients. Patients were categorized into three groups based on the mean HK2 levels and MT-RNR1 G709A status in HCC tumors, with Group I consisting of 17 patients with low HK2 and MT-RNR1 709G, Group II of 16 patients with low HK2 and MT-RNR1 709A or high HK2 and MT-RNR1 709G, and Group III of 9 patients with high HK2 and MT-RNR1 709A. Patients in Group III showed significantly reduced overall survival compared to patients in Groups I and II (Figure 5F). Taken together, our findings indicate that the MT-RNR1 G709A and HK2 expression levels are potential prognostic biomarkers in patients with HCC.

## 3. Discussion

Previous studies identified that multiple point mutations in the mitochondrial genome were highly associated with cancer progression [31]. The majority of mutations were identified in the D-loop region of the mitochondria genome [32]. Notably, the MT-RNR1 A1555G mutation is associated with aminoglycoside-induced and nonsyndromic sensorineural deafness hearing loss [11,33]. Point mutations in the mtDNA D-loop 310C (noncoding region) [34,35], G709A (noncoding region) [36], C12705T (MT-ND5) [36], and A8701G (MT-ATP6) [37] have been identified by other groups. Accordingly, the single-nucleotide polymorphisms (SNPs) in mtDNA could be a useful biomarker for the prediction of disease and cancer progression. However, the correlation between MT-RNR1 G709A and liver metastasis remains unclear. In the current study, we provide evidence that a specific mtDNA variation, MT-RNR1 G709A, contributes to the risk of metastatic HCC.

MOTS-c is a small functional peptide (16 amino acids) encoded within the MT-RNR1. Previous studies have indicated that MOTS-c modulates insulin resistance and cellular metabolism through the activation of AMP-activated protein kinase [27]. Lu et al. [38] demonstrated that MOTS-c is a therapeutic agent to protect cells against cold stress. Another study showed that MOTS-c treatment represses vascular calcification in a rat model [29]. Accordingly, MOTS-c regulates many aspects of metabolism and physiology. However, the functional role of MOTS-c in HCC was still unclear. Here, we found that MOTS-c treatment in hepatoma cell lines suppressed cells’ migratory ability. Furthermore, MT-RNR1 709A downregulated MOTS-c expression, which in turn resulted in inducing cell metastasis.

Tumor cells can survive in some specific microenvironments, such as hypoxia or metabolic stress condition via the regulation of their own cellular metabolism [39]. Notably, hepatoma cells prefer to regulate glucose metabolism [40]. Another study indicated that the knockdown of HK2 in hepatoma cells suppressed tumor formation via the modulation of glycolysis and oxidative phosphorylation [41]. Recently, our group identified that taurine upregulated gene 1 (TUG1) promotes cancer cell glycolysis and metastasis through the regulation of HK2 [42]. These investigations indicate that glycolytic-related gene, HK2, is upregulated in HCC and associated with survival outcomes. Another study demonstrated that mutations were identified in noncoding regions of the mitochondrial genome that contain key regulatory elements, such as promoters [43]. Here, we found that the HK2 expression in the MT-RNR1 709A group was higher than that in the MT-RNR1 709G group. Meanwhile, patients with high HK2 and MT-RNR1 709A exhibited reduced overall survival compared to patients in other groups. These findings suggest that HK2 expression is regulated by distinct MT-RNR1 G709A. Taken together, we propose that MT-RNR1 G709A may favor glycolytic flux, but not mitochondrial OXPHOS in hepatoma. However, the underlying mechanism of their association should be further investigated in the future.

## 4. Materials and Methods

### 4.1. Human HCC Samples

Our major goal was to identify an mtDNA sequence to predict a poor prognosis in patients with metastatic HCC. Patients in the training cohort were divided into patients in a non-metastatic group (*n* = 16) and metastatic group (*n* = 9). We analyzed the mtDNA sequence of each patient and compared them between the two groups in the training cohort. To verify the mtDNA sequence established from the training cohort, a total of 200 subjects were included in a validation cohort. The clinical parameters of the patients enrolled are shown in Supplementary Table S5, including age, gender, cirrhosis, AFP, viral status, bilirubin (Bil), ALT, AST, PT, alcoholism, tumor number, largest tumor size, Edmondson histology grade, microvascular invasion, and macrovascular invasion. Tumor liver tissues were resected at Chang Gung Memorial Hospital after informed consent was obtained. This study was approved by the Ethics Committee of Chang Gung Memorial Hospital.

### 4.2. Mutation Analysis of Entire Mitochondrial Genome

mtDNA was extracted from HCC specimens, and mtDNA sequencing was used to investigate mtDNA mutations. Overlapping DNA fragments were generated by polymerase chain reaction (PCR) amplification spanning the entire mitochondrial genome. PCR was performed for 30 cycles in a 50 μL reaction mixture containing 100 ng DNA, 200 μM of each dNTP, 2.5 μM of each primer, DNA polymerase (BIOTOOLS CO., LTD. Taiwan), and 10× reaction buffer (BIOTOOLS). PCR cycles consisted of 30 s denaturation at 94 °C, 30 s annealing at 58 °C and 1 min primer extension at 72 °C. Direct DNA sequencing was performed from both directions for all samples. Sequence variants identified from both directions of sequencing were completely matched. The Mitomap (http://www.mitomap.org) and mtDB (http://www.genpat.uu.se/mtDB/) databases were used to identify sequence variants.

### 4.3. Immunoblot Analysis

The immunoblot procedure was performed as described previously. Antibodies specific for HK2 (Cell Signaling Technology, Boston, MA, USA), ATP6 and CTYB (Abcam, Cambridge, MA, USA), COXIV-1 (Thermo Fisher Scientific Inc., Kalamazoo, MI, USA), and ß-actin (Sigma-Aldrich, St Louis, MO, USA) were used. The signals of the target genes were analyzed using the Image Gauge software (Fujifilm, Tokyo, Japan) and further normalized to those of β-actin.

### 4.4. Establishment of HK2 Overexpression or Knockdown Stable Cell Lines

Full-length HK2 sequences were cloned into pcDNA3 vector (Invitrogen, Carlsbad, CA, USA). The pool of stably transfected cells was selected in medium containing neomycin. Specific four shRNAs (TRCN0000037671, TRCN0000037672, TRCN0000196260, and TRCN0000196724) for the HK2 gene were purchased from the National RNA Interference Core Facility (Institute of Molecular Biology, Academia Sinica, Taiwan). The shRNAs of HK2 used in this study are shown in Supplementary Table S6. Single shRNA plasmid and virus package plasmids (pCMV-△R8.91 and pMD.G) were co-transfected into 293 cells and the virus was harvested after 48 h. A pool of stably infected cells was selected in medium containing puromycin. The expression levels of HK2 in stable cell lines were validated by Western blot analysis.

### 4.5. In Vitro Migration Assay

Cell migratory activities were detected by an in vitro Transwell assay (Becton-Dickinson, Franklin Lakes, NJ, USA) using inserts with a membrane pore size of 8 mm. Cell number was adjusted to 500 cells/mL, and 100 μL suspension was seeded on the upper chambers of the Transwell plate (Becton-Dickinson). After incubation for 16 h at 37 °C, cells traversing the filter from the upper to the lower chamber were determined via crystal violet staining. Migratory cells were calculated by the Image J software.

### 4.6. Statistical Analysis

Statistical analysis was performed in SPSS version 20 (SPSS Inc., Chicago, IL, USA) using the Mann–Whitney test for the comparison of two groups and a one-way ANOVA followed by Tukey’s post-hoc test for two or more groups. Kaplan–Meier survival curves were employed to analyze survival outcomes. Overall survival with death as an event was analyzed using the log-rank test. *p* values < 0.05 were considered significant.

## 5. Conclusions

In conclusion, for the first time our results show the importance of the mtDNA G709A variation and its correlation with highly metastatic hepatoma. Notably, combined MT-RNR1 G709A and increased expression of HK2 was a stronger predictor of overall survival in patients. Thus, understanding the cross-talk between glycolysis and mtDNA polymorphisms and how they influence each other in all types of cancers is an important concept in cancer biology and molecular biology.

## Data Availability

The data presented in this study are available on request from the corresponding author.

## Supplementary Materials

Supplementary Materials can be found at https://www.mdpi.com/1422-0067/22/3/1119/s1.

## Abbreviations

mtDNA	mitochondrial DNA
HCC	hepatocellular carcinoma
OXPHOS	oxidative phosphorylation
ATP6	mitochondrially encoded ATP synthase membrane subunit 6
ND	NADH dehydrogenase
ROS	reactive oxygen species
NSCLC	non-small cell lung cancer
cybrid	cytoplasmic hybrid
SNP	single nucleotide polymorphism
AFP	alpha-fetoprotein
Bil	bilirubin
ALT	alanine transaminase
AST	aspartate transaminase
PT	prothrombin time
qRT-PCR	quantitative real-time polymerase chain reaction
HR	hazard ratio
HK2	hexokinase 2
MOTS-c	mitochondrial open-reading-frame of the twelve S rRNA-c
TUG1	taurine upregulated gene 1
