# Supplementary material for: Impact of an MT-RNR1 Gene Polymorphism on Hepatocellular Carcinoma Progression and Clinical Characteristics
Source: Int J Mol Sci. 2021 Jan 23;22(3):1119. doi: 10.3390/ijms22031119 (PMC7865300; doi:10.3390/ijms22031119)
Supplement: Supplementary file 1 [file ijms-22-01119-s001.pdf]

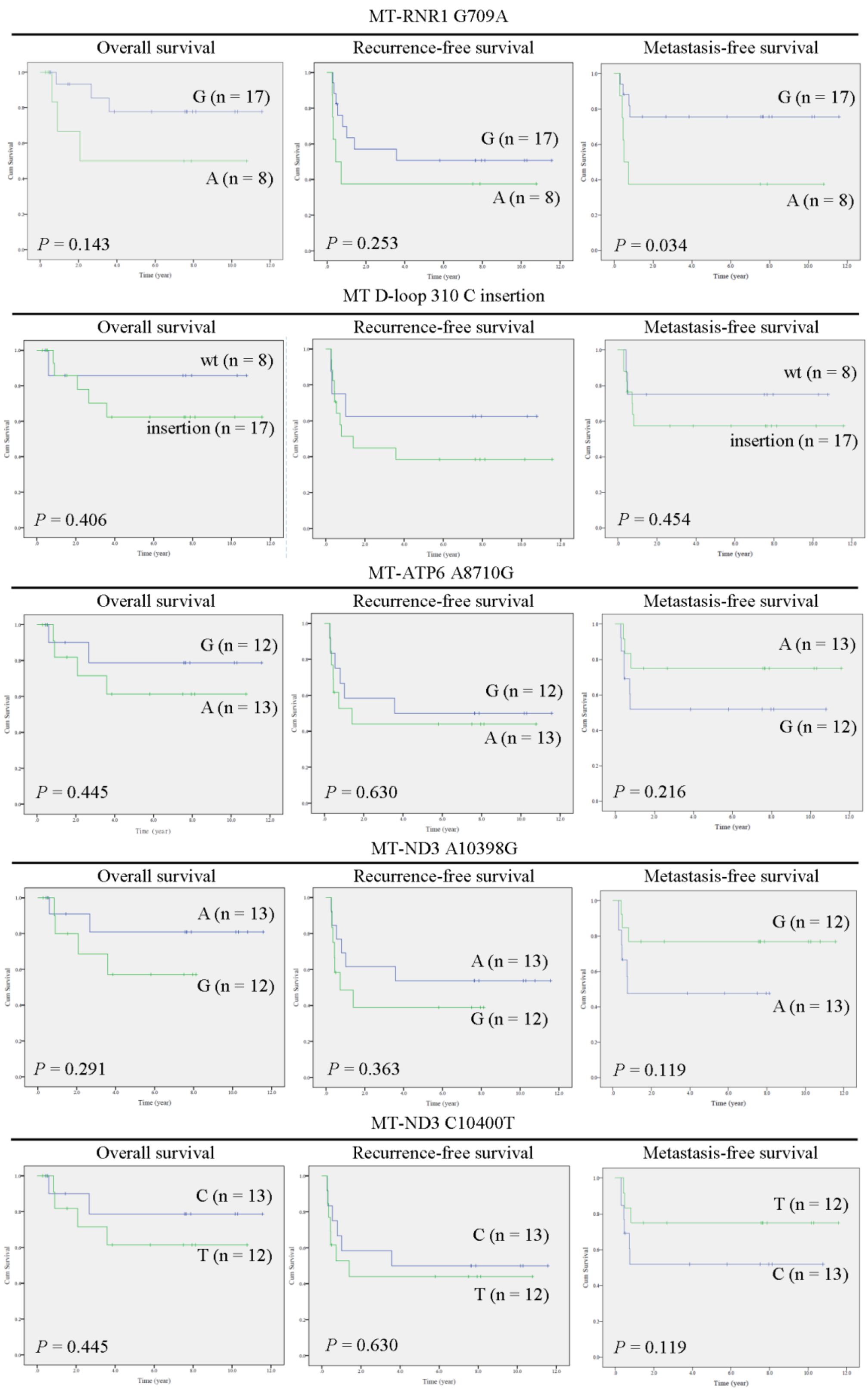

Supplementary Figure 1. Survival outcome based on mitochondrial mutations. Kaplan-Meier analysis of survival outcomes based on these mutations in training cohort. Survival function was analyzed using the log-rank test.

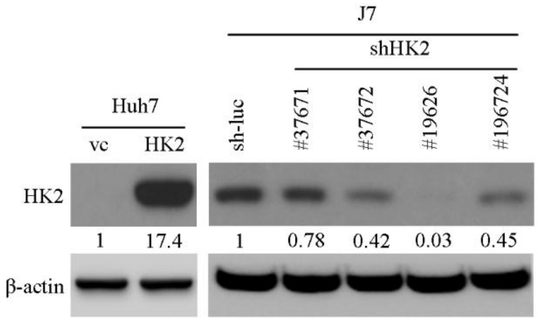

Supplementary Figure 2. HK2 stable cell lines were established.

Expression of HK2 in the indicated cell lines were determined by Western blot analysis. β-actin was used as loading control. The quantitative result was shown. Vc: vector control.

Supplementary Table 1. Mitochondrial DNA variants detected in hepatocellular carcinoma

type	Gene name/Location	location	SNP	Amino acid change
Non-coding	D-loop	310	C insertion	NA[a]
Non-coding	MT-RNR1	709	G-A	NA[a]
Protein-coding	ATP6	8701	A-G	Thr→Ala
Protein-coding	ND3	10398	A-G	Thr→Ala
Protein-coding	ND3	10400	C-T	Thr→Ala

[a] NA, not applicable.

Supplementary Table 2. Clinicopathological correlation of mtDNA sequences in human HCC

Clinicopathological Features		MT-RNR1		*P* Value
		wt (G)	SNP (A)	
Gender	Male	119	37	0.6921
	Female	32	12	
Age	< 65	111	30	0.1081
	≧ 65	40	19	
HBV	Present	119	33	0.1238
	Absent	32	16	
HCV	Present	42	14	1.0000
	Absent	109	35	
Cirrhosis	Present	90	28	0.8674
	Absent	61	21	
AFP, ng/ml	< 400	108	35	1.0000
	≧ 400	43	14	
Bil, mg/dl	< 1.2	121	35	0.2344
	≧ 1.2	30	14	
PT, sec	< 12	81	29	0.5140
	≧ 12	70	20	
AST, U/L	< 31	48	13	0.5929
	≧ 31	103	36	
ALT, U/L	< 41	70	25	0.6230
	≧ 41	81	24	
Alcohol	Positive	48	10	0.1492
	Negeative	103	39	
Tumor size, cm	< 5	73	23	0.8711
	≧ 5	78	26	
Grade	1-2	53	21	0.3948
	3-4	98	28	
Microvascular invasion	Present	40	17	0.2792
	Absent	111	32	
Macrovascular invasion	Present	15	6	0.7907
	Absent	130	43	

Supplementary Table 3. Univariate and multivariate analyses of overall survival in HCC patients by Cox regression analysis

	Univariate Analysis		Multivariate analysis	
Variables	HR (95% CI)	*P*	HR (95% CI)	*P*
Gender	0.662 (0.251-1.742)	0.403		
Age	1.768 (0.694-4.504)	0.232		
Cirrhosis	0.703 (0.285-1.731)	0.444		
HBV	0.622 (0.236-1.641)	0.338		
HCV	1.224 (0.465-3.224)	0.682		
AFP	1.946 (0.782-4.843)	0.152		
Bil	2.452 (0.965-6.235)	0.060		
ALT	0.929 (0.377-2.288)	0.873		
AST	3.870 (0.894-16.761)	0.070		
PT	1.060 (0.430-2.611)	0.900		
Alcohol	1.217 (0.462-3.204)	0.691		
Tumor size	2.867 (1.085-7.578)	0.034	2.964 (1.120-7.845)	0.029
Grade	0.986 (0.495-1.965)	0.968		
Microvascular invasion	1.411 (0.535-3.717)	0.487		
Macrovascular invasion	1.235 (0.284-5.366)	0.778		
MT-RNR1 G709A	3.986 (1.617-9.829)	0.003	4.097 (1.659-10.114)	0.002

Abbreviations: HR: hazard ratio; CI: confidence interval

Supplementary Table 4. Univariate and multivariate analyses of metastasis-free survival in HCC patients by Cox regression analysis

	Univariate Analysis		Multivariate analysis	
Variables	HR (95% CI)	*P*	HR (95% CI)	*P*
Gender	2.512 (0.580-10.889)	0.451		
Age	0.963 (0.346-2.681)	0.942		
Cirrhosis	0.410 (0.160-1.053)	0.064		
HBV	1.142 (0.379-3.442)	0.814		
HCV	0.734 (0.243-2.218)	0.584		
AFP	1.525 (0.600-3.874)	0.118		
Bil	0.884 (0.256-3.052)	0.846		
ALT	0.793 (0.322-1.953)	0.614		
AST	1.292 (0.465-3.587)	0.623		
PT	1.855 (0.719-4.788)	0.201		
Alcohol	1.511 (0.594-3.843)	0.386		
Tumor size	2.082 (0.817-5.306)	0.124		
Grade	0.696 (0.349-1.388)	0.303		
Microvascular invasion	2.388 (0.958-5.953)	0.062		
Macrovascular invasion	3.073 (1.018-9.275)	0.046	3.360 (1.106-10.209)	0.033
MT-RNR1 G709A	2.603 (1.046-6.478)	0.040	2.758 (1.104-6.889)	0.030

Abbreviations: HR: hazard ratio; CI: confidence interval

Supplementary Table 5. Basic clinicopathological factors of patients included

Variables	Patient numbers
Age (years)	
＜65	141 (70.5%)
≧65	59 (29.5%)
Gender	
Male	156 (78%)
Female	44 (22%)
Cirrhosis	
No	82 (41%)
Yes	118 (59%)
AFP	
< 400 ng/ml	143 (71.5%)
≧ 400 ng/ml	57 (28.5%)
Viral status	
NBNC	13 (6.5%)
HBV	131 (65.5%)
HCV	35 (17.5%)
HBV+HCV	21 (10.5%)
Bilirubin	
＜ 1.2 mg/dL	156 (78%)
≧ 1.2 mg/dL	44 (22%)
ALT	
＜ 41 IU/L	95 (47.5%)
≧ 41 IU/L	105 (52.5%)
AST	
＜ 31 IU/L	61 (30.5%)
≧ 31 IU/L	139 (69.5%)
Prothrombin time	
＜ 12 sec	110 (55%)
≧ 12 sec	90 (45%)
Alcohol	
Negative	142 (71%)
Positive	58 (29%)
Tumor number	
1	106 (53%)
> 1	94 (47%)
Tumor size	
＜ 5 cm	96 (48%)
≧ 5 cm	104 (52%)
Grade	
1-2	74 (37%)
3-4	126 (63%)
Microvascular invasion	
Negative	143 (71.5%)
Positive	57 (28.5%)
Macrovascular invasion	
Negative	173 (86.5%)
Positive	21 (10.5%)

Supplementary Table 6. HK2 shRNA sequences used in this study were listed.

Name (Clone ID)	target sequence
shHK2#1 (TRCN0000037672)	CACTGTGAAGTTGGCCTCATT
shHK2#2 (TRCN0000196260)	GCTTGAAGATTAGGTACTATC
shHK2#3 (TRCN0000037671)	ACTGAGTTTGACCAGGAGATT
shHK2#4 (TRCN0000196724)	GACTTTGATATCGACATTGTG
